# Exploring Cannabidiol (CBD) and Cannabigerol (CBG) Safety Profile and Skincare Potential

**DOI:** 10.3390/ijms252212224

**Published:** 2024-11-14

**Authors:** Mariana Luz-Veiga, Adélia Mendes, Diana Tavares-Valente, Manuela Amorim, António Conde, Manuela Estevez Pintado, Helena R. Moreira, João Azevedo-Silva, João Fernandes

**Affiliations:** 1 CBQF—Centro de Biotecnologia e Química Fina—Laboratório Associado, Escola Superior de Biotecnologia, Universidade Católica Portuguesa, Rua Diogo Botelho 1327, 4169-005 Porto, Portugal; mveiga@ucp.pt (M.L.-V.); marmendes@ucp.pt (A.M.); dvalente@ucp.pt (D.T.-V.); mamorim@ucp.pt (M.A.); mpintado@ucp.pt (M.E.P.); hrmoreira@ucp.pt (H.R.M.); jasilva@ucp.pt (J.A.-S.); 2 Amyris Bio Products Portugal, Unipessoal Lda, Rua Diogo Botelho 1327, 4169-005 Porto, Portugal; 3 Hospital Lusíadas, Avenida da Boavista 171, 4050-115 Porto, Portugal

**Keywords:** cannabidiol, cannabigerol, safety testing, cosmetics, skincare, mutagenicity, skin sensitization, cytotoxicity

## Abstract

Cannabinoids have long been known for their bioactive properties, with their topical application as anti-inflammatory compounds being at the forefront of research for the past decade. Concurrently, the cosmetic market is a fast-growing industry in constant need of new biomolecules. In this work, we studied the safety profile for topical applications of two cannabinoids: cannabidiol (CBD) and cannabigerol (CBG) and assessed their potential as skin care ingredients. The CBG used in this work resulted from bio-fermentation, and to the best of our knowledge, there are no extensive reports on its safety and usage as a cosmetic ingredient. Our results show that CBD and CBG do not exhibit cytotoxicity, mutagenicity, or skin sensitization. Moreover, we verified an absence of primary irritability, accumulated irritability, phototoxicity and photosensitization, supporting the claims of dermatologically tested, hypoallergenic and non-irritating. While these cannabinoids did not show significant anti-aging effects by altering the extracellular matrix components (both in vitro and ex vivo), they demonstrated promise as protective agents against inflammation caused by air pollution. Specifically, they reduced the levels of pro-inflammatory cytokines, making them valuable in combating environmental skin damage. Overall, our results validate the safety of topical use of cannabinoids, while paving the way for further research in the beauty and personal care market as soothing agents.

## 1. Introduction

The skin is the largest organ of the human body, creating the first barrier against chemical, physical and biological agents. It is composed of the epidermis, the dermis and adipose tissue, each with distinctive functions and characteristics [1]. While the epidermis exerts a barrier function, particularly against UV radiation, the dermis contributes to sustaining the elasticity and mechanical integrity of the skin, comprising three major cell types contained in an extracellular matrix (ECM) [1,2]. Skin changes occur in a time-dependent manner and are modulated by environmental factors. Among these, sun exposure is among the most relevant external factors affecting the skin’s ECM, leading to skin aging [3]. This process is predominantly characterized by the thinning and wrinkling of the epidermis, due to changes in the dermis resulting from the reduction in collagen and elastin fibers, both proteins belonging to the ECM. This results in the impairment of the support of the epidermal layer [4].

Cosmetic formulations containing nature-derived bioactive ingredients can protect the skin against harmful exogenous and endogenous agents, improving various skin conditions (e.g., redness, dryness, itchiness) and reducing changes induced by extrinsic and intrinsic aging mechanisms by enhancing the renewal of dermal ECM components. In recent decades, consumer demand for cosmetic formulations with natural ingredients has increased significantly [5], leading to high commercial value [6]. This trend aligns with the significant growth in the global skincare market. However, concerns about the secondary effects of cosmetics and personal care products are also rising. The irritant potential of an ingredient is influenced by several factors, including absorption, concentration, and cumulative effect. As a result, cosmetic companies utilize various assays to assess the safety of their products for topical application. In Europe, the framework for the safety of any cosmetic product is well-established. Different steps are taken to assess if a molecule is genotoxic, a skin sensitizer, or comedogenic, among other characteristics detailed elsewhere [7]. Several other tests are used to ensure the safety of cosmetics on the skin, including patch tests to evaluate an irritant or allergic reaction, and phototoxicity and photosensitization tests to determine whether a product can cause skin reactions when exposed to light.

Phytocannabinoids stand out as an example of potential bioactive/cosmetic ingredients. Since their initial isolation in the 1940s, these molecules have been associated with a myriad of biological activities, including anti-inflammatory, antioxidant, and antimicrobial properties [8,9,10]. Thus far, over 120 cannabinoids have been identified and isolated from *Cannabis sativa* L. Notwithstanding, most studies focus solely on cannabidiol (CBD) and tetrahydrocannabinol (THC), as these are the two phytocannabinoids found in the highest concentrations in the Cannabis plant [11]. Other phytocannabinoids, such as cannabigerol (CBG) and cannabichromene (CBC), are considered minor cannabinoids, as their yield tends to be inferior to 1% [12]. To further explore the bioactive potential of cannabinoids, the development of synthetic cannabinoids and the production of cannabinoids via yeast fermentation has been implemented [13,14]. Initial reports suggest that these cannabinoids might have a wide range of benefits in the treatment of different skin conditions, such as acne and eczema, similar to what has been observed for CBD and THC [12]. Such effects are attributed to their anti-inflammatory, antioxidant, and moisturizing properties. Particularly, in recent years there has been an increase in the marketed CBD and CBG-containing formulations for both dermatological and cosmetics purposes, with claims pertaining to CBD’s capacity to improve skin hydration [15], reduce acne and aging consequences [16], and modulate hair growth [17]. Moreover, considering their antimicrobial effect, CBD and CBG’s effect as cosmetic formulation preservatives has also been studied, successfully preventing microbial development and demonstrating a lack of impact on the skin microbiota [10]. However, many of the CBD-containing formulations currently on the market make unsubstantiated claims about their beneficial properties for the skin [18]. Thus, it is necessary to assess the safety and current evidence on the use of cannabinoids for cosmetic purposes.

Considering the aforementioned properties, we hypothesized that CBD and CBG could have potential as cosmetic ingredients due to their safety and bioactivity. Hence, we aimed to assess if CBD and CBG are safe for topical application and if they demonstrated any relevant effect as potential cosmetic ingredients. To do so, we first studied CBD and CBG’s safety for skin application, addressing different key parameters, such as cytotoxicity, genotoxicity, skin sensitization and phototoxicity. This is particularly relevant for the CBG used, as it is derived from bio-fermentation, with no reports on its safety yet. Additionally, we investigated their antioxidant capacity, as well as their impact on cosmetically relevant skin markers (e.g., production of collagen and hyaluronic acid, among others), using in vitro and ex vivo experimental models.

## 2. Results

### 2.1. Cannabinoids Are Safe at Tested Concentrations

To ensure the safety of using CBD and CBG for improving skin health through topical application, various Organization for Economic Cooperation and Development (OECD) tests were conducted to assess the potential for these cannabinoids to cause skin sensitization, as the OECD Test Guidelines are recognized as standards for safety testing of chemicals and chemical products. The results are summarized in Table 1, which indicates that there was no observed sensitizing potential for the tested cannabinoids at the lowest concentrations. Interestingly, while CBD showed sensitizing potential at 25 μM, CBG had negative sensitizing results for both concentrations tested. Cinnamic aldehyde was used as a positive control, attaining a sensitizing potential over 15 times higher than both cannabinoids.

Regarding the h-CLAT, as seen in Table 2, none of the tested cannabinoids had a potential sensitizing effect at any of the concentrations tested, with a cell viability percentage superior to 80%. The concentrations of cannabinoids to be tested were established through a viability assay (Supplementary Figure S1) and further calculation of the CV75. Both cannabinoids had similar results, with RFI values ranging from 76 to 144 for CD54 and from 75 to 132 for CD86 and, thus, never surpassing the positive criteria. Nickel(II) sulfate was used as a positive control.

Next, we addressed the genotoxic potential of CBD and CBG. As seen in Table 3 and Table 4, these cannabinoids do not have any potential genotoxicity potential at the concentrations tested as seen through the AMES fluctuation test, since there was not a significant increase in the number of revertants.

**Figure 1:**
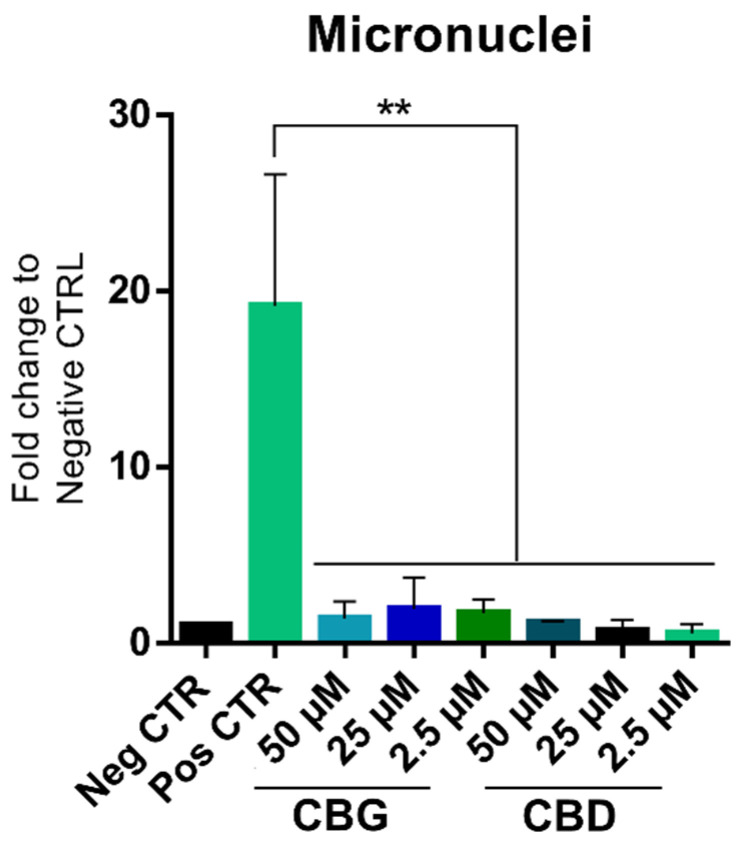
Results of the micronucleus assay, as described in OECD guideline 487. The data were obtained from three independent experiments with three replicates for each condition. ** *p* < 0.01; one-way ANOVA coupled with Tukey’s post hoc test.

The micronuclei assay results for both CBD and CBG indicate that neither of these cannabinoids induced a significant development of micronuclei in the tested conditions (Figure 1). These findings are consistent with the absence of genotoxicity, as they suggest that CBD and CBG did not cause damage to the chromosomes or DNA in the tested cells. The use of Mitomycin C as a positive control, which led to a significant increase in micronuclei formation, confirms the sensitivity of the assay (Supplementary Figure S2). Therefore, CBD and CBG appear to be safe in terms of mutagenicity potential under the conditions tested.

**Figure 2:**
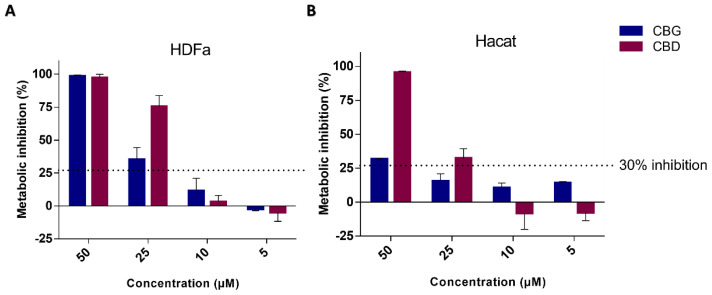
Results of the biocompatibility assay for HDFa (**A**) and HaCaT (**B**) exposed to cannabinoids. The data are represented as the mean ±  SD for *n* = 3 independent experiments. A cytotoxic effect is assumed for a metabolic inhibition exceeding 30%, in accordance with ISO 10993-5.

In addition to mutagenicity and skin sensitization tests, the safety of cannabinoids for skin application was studied through cytotoxicity against skin-derived cell lines (Figure 2A,B). For that, the impact of cannabinoids on HaCaT and HDFa metabolic activity was evaluated, with CBG demonstrating a lower value of metabolic inhibition for HDFa and HaCaT than CBD. A control of the solvent used was performed (ethanol 1% (*v*/*v*)), and no inhibition was demonstrated.

Additionally, the irritant potential of both CBD and CBG was assessed through patch tests. As shown in Table 5, none of the research participants experienced any significant cutaneous reactions (such as erythema, edema, papules, or vesicles) at the sites where the cannabinoids were applied, whether for primary or accumulated irritability, phototoxicity, or photosensitization, during the study period. There were no reports or evidence of adverse reactions, supporting the claims that these molecules are dermatologically tested, hypoallergenic, and non-irritating. Further details can be found in Supplementary Tables S1–S3.

Given the widespread use of CBD in topical applications, the lack of sensitizing potential, mutagenicity, or cytotoxicity is expected. However, there is limited information available on the safety profile of CBG. These initial results indicate that both molecules are safe for topical application.

### 2.2. Cannabinoids Have Low Antioxidant Capacity

**Figure 3:**
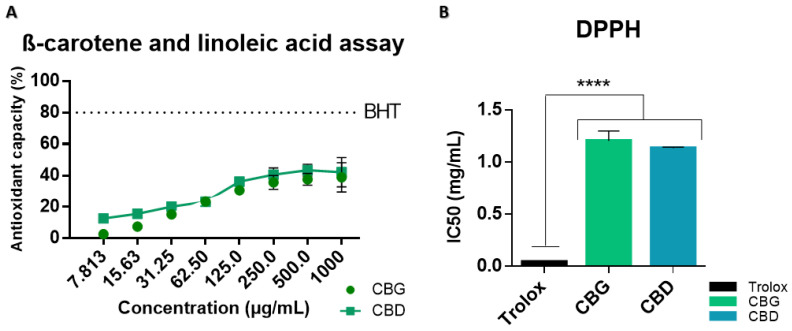
Results of the (**A**) β-carotene and linoleic acid assay using BHT as a positive control. (**B**) DPPH assay with Trolox as a positive control. The data were obtained from three independent experiments with three replicates for each condition. **** *p* < 0.0001; one-way ANOVA coupled with Tukey’s post hoc test.

Studies have shown that cannabinoids have a strong antioxidant capacity [19]. However, our results demonstrated a lack of antioxidant capacity for both cannabinoids tested, especially when compared with the antioxidant controls (Figure 3).

The β-carotene and linoleic acid assay is based on the bleaching of β-carotene due to its reaction with radicals, resulting in the oxidation of linoleic acid. However, the loss of color can be reduced in the presence of antioxidants. Cannabinoids presented modest antioxidant capacity (Figure 3A), as the maximum antioxidant capacity achieved did not surpass 50%, with the curve reaching a plateau at 500 μg/mL. On the contrary, the positive control BHT attained an 80% antioxidant capacity and was significantly (*p* < 0.05) higher than all samples tested. Regarding the DPPH assay (Figure 3B), it is routinely used to assess the antioxidant capacity of plant extracts, determining if a molecule reacting with a stable free radical DPPH• can scavenge it, resulting in its discoloration. This assay further supported the previous results, with cannabinoid IC50 values significantly lower than the positive control (Trolox), demonstrating a lower protective effect/antioxidant capacity.

### 2.3. Cannabinoids Do Not Prevent ROS Generation but Have Anti-Inflammatory Effect

**Figure 4:**
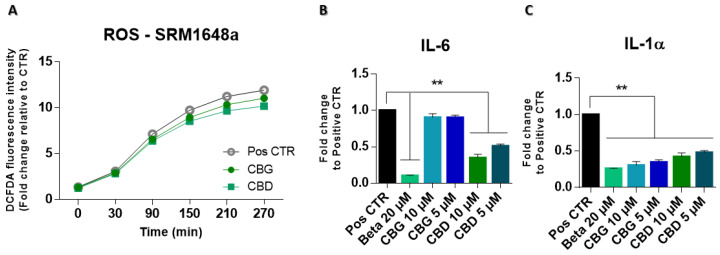
Cannabinoids were unable to significantly prevent the generation of ROS in the presence of UPM but were able to reduce pro-inflammatory cytokines levels in the same context. (**A**) Results of the ROS assay using the DCFDA probe. ELISA results of IL-6 (**B**) and IL-1α (**C**) levels for keratinocytes exposed to UPM. Results are presented as a fold change to the basal condition (Pos CTR—cells with UPM), with data normalized to cytokine concentration per mg of protein. Betamethasone (Beta 20 μM) was used as an anti-inflammatory control. Data were obtained from three independent experiments with two replicates for each condition. ** *p* < 0.01, determined by one-way ANOVA coupled with Tukey’s post hoc test.

As CBD and CBG have anti-inflammatory activity, and exposure to pollution is known to lead to an inflammatory response and oxidative stress, we assessed whether both molecules were able to mitigate ROS generation and prevent the production of pro-inflammatory cytokines. As seen in Figure 4A, cannabinoids were unable to significantly inhibit ROS generation in cells exposed to UPM, with no significant differences over time for either CBD or CBG, compared to the positive control. In addition to ROS generation, it was important to assess whether cannabinoids could reduce the inflammation commonly associated with exposure to pollution. As seen in Figure 4B,C, CBD was able to significantly reduce IL-6 and IL-1α levels. Interestingly, CBG was unable to reduce IL-6 levels, yet it significantly reduced IL-1α amounts.

### 2.4. Cannabinoids Do Not Stimulate the Production of ECM Component In Vitro and Ex Vivo

Several enzymes contribute to the skin’s homeostasis, and imbalances in their activity might lead to different skin disorders. Therefore, the potential of cannabinoids to inhibit the activity of enzymes with cosmetic relevance (i.e., skin aging enzymes) was assessed (Table 6). Here, we studied collagenase (MMP-1) and elastase, which are responsible for ECM degradation, and tyrosinase, which contributes to hyperpigmentation. Overall, none of the cannabinoids tested exerted inhibitions similar to the inhibition controls. For instance, CBD was able to inhibit MMP-1’s activity by more than 60%, while not affecting the activity of elastase (37%) and tyrosinase (38%). As for CBG, we observed that it was able to significantly inhibit MMP1 (59%), tyrosinase (87%) and elastase (57%).

**Figure 5:**
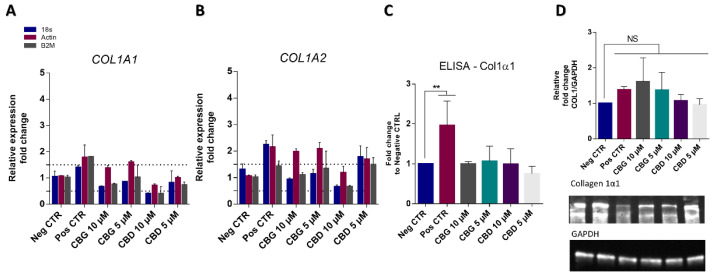
Cannabinoids did not impact the expression of collagen in human dermal fibroblasts, as seen through genetic and protein expression. Results of qPCR with COL1A1 (**A**) and COL1A2 (**B**), using three housekeeping genes. The dotted line represents 0.5-fold higher and lower than the basal level. Collagen levels were also assessed by ELISA (**C**) and Western blot (**D**). No statistical differences (NS) were found between the negative control and the cannabinoid samples for the Western blot samples. Pal-GHK was used as a positive control for collagen production (Pos CTR). Data were obtained from three independent experiments with two replicates for each condition. ** *p* < 0.01, determined by one-way ANOVA coupled with Tukey’s post hoc test.

We next assessed the impact of cannabinoids on the gene and protein expression of different ECM components, including collagen type 1 (Figure 5) and fibronectin (Supplementary Figure S3) in cultured dermal fibroblasts as well as in human skin ex vivo models. Our results show that none of the tested cannabinoids were able to significantly (*p* > 0.05) alter collagen type I levels in cultured cells, both at an mRNA level (Figure 5A,B) and protein level (Figure 5C,D). Pal-GHK, a peptide known for its anti-aging effects and stimulation of ECM production, was used as a positive control for collagen production.

**Figure 6:**
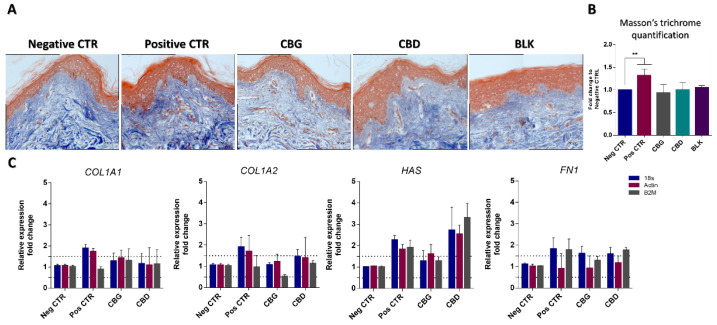
Ex vivo results for Masson’s trichrome staining (**A**) and respective quantification (**B**). Collagen is stained blue, nuclei are stained purple, and cytoplasm is stained red. No statistical differences were found between the negative control and the cannabinoid samples. The scale bars for all images are 20 μm. (**C**) Results for qPCR with *COL1A1*, *COL1A2*, *FN1* and *HAS1* with three housekeeping genes. The dotted line represents 0.5-fold higher and lower than the basal level. A significant (*p* < 0.01) increase in *HAS1* expression was observed for CBD. Matrixyl was used as a positive control (Pos CTR). Data were obtained from three independent experiments with two replicates for each condition. ** *p* < 0.01, determined by one-way ANOVA coupled with Tukey’s post hoc test.

The expression of additional ECM components (aquaporin 3, hyaluronic acid and elastin) in response to CBD and CBG exposure was studied by qPCR and ELISA, with no significant variations compared to the control (untreated cells) at any given concentration (Supplementary Figure S4). Although no significant variations were found for the tested cannabinoids in our in vitro cellular models, we decided to extend our studies to their potential use in topical formulations using skin explants. Ex vivo models are composed of several functioning cellular layers, thus translating into a more complex environment than 2D cell culture models. The results of the ex vivo antiaging assay are presented in Figure 6. The quantification of Masson’s trichrome staining shows that neither CBD nor CBG treatment led to significant changes in collagen fiber levels. Moreover, none of the tested cannabinoids was able to induce significant changes in the expression of ECM-related genes, except for a significant increase in the gene expression of hyaluronan synthase (*HAS1*) for CBD. Matrixyl 3000 was used as an anti-aging control and significantly (*p* > 0.05) increased collagen levels in the ex vivo model.

To assess whether the lack of anti-aging properties could be a result of cannabinoids not permeating the skin barrier, skin permeation studies were conducted. Several physicochemical properties are required to facilitate passive absorption and permeation through the epidermis. These include a molecular weight below 500 g/mol, moderate lipophilicity (octanol–water partition coefficient of 1–4), adequate lipid and aqueous solubilities, and finally, relatively low solubility in the vehicle (if any) and low volatility. Considering that both CBD and CBG meet these criteria (i.e., CBD and CBG have molecular weights of 314 and 316 g/mol, respectively, and both possess moderate lipophilicity (6.3 for CBD and 7.1 for CBG), their permeation through the epidermis should be facilitated. Our results demonstrated that both CBD and CBG had an absorbed dose of approximately 70% (Table 7). A range of 25 to 30% remained on the skin, while less than 5% did not permeate the epidermis. Thus, the absence of effects by cannabinoids is not attributed to permeation issues.

## 3. Discussion

Considering the widespread usage of CBD and other cannabinoids in topical applications, the absence of sensitizing potential, mutagenicity, and cytotoxicity is expected. However, to the best of our knowledge, this is the first detailed report on the skin sensitization potential of CBD and CBG following OECD guidelines, and the first report concerning the irritation potential of CBG in vivo. It was essential to establish the safety profile of CBG, as it was produced via bio-fermentation and its safety had yet to be assessed. No irritation was induced by neither CBG nor CBD, as no significant skin sensitization, photosensitivity, or phototoxicity were detected during the study period. While reports concerning CBG are scarce, its absence of phototoxicity has been demonstrated, as well as its lack of induced-irritation in rabbit skin [20,21]. Moreover, the lack of sensitization for CBD is in accordance with reports by Maghfour, et al. [22] who demonstrated that CBD alone or in a hemp seed oil formulation did not result in skin irritation nor sensitization via patch testing on healthy human skin. Thus, the *in chemico*, in vitro, and in vivo results show that both molecules can be considered safe for topical application.

After validating their safety profile, we further investigated the antioxidant capacity of CBG and CBD, which is a key parameter in the development of cosmetic ingredients. Cosmetic ingredients with a significant antioxidant capacity can, for example, scavenge ROS, inhibit enzymes responsible for collagen degradation and, consequently, reduce aging effects. However, the DPPH and β-carotene/linoleic acid assays showed that CBD and CBG exhibit modest antioxidant activity only, which was significantly inferior to the positive controls tested. This is not in accordance with reports about CBD’s antioxidant capacity as assessed by the DPPH assay [23,24]. These differences may be explained by the different concentrations assayed and the changes to the DPPH protocol.

Although neither cannabinoid demonstrated significant antioxidant capacity, we chose to assess whether CBD and CBG could prevent oxidative stress in keratinocytes. These cells are in direct contact with air pollution, resulting in inflammation and oxidative stress [25]. Therefore, we studied the antioxidant and anti-inflammatory activity of cannabinoids when HaCaT cells are exposed to urban air pollution. In contrast to previous reports, we did not observe a direct inhibition of ROS generation by CBG or CBD. Nevertheless, it is worth noting that these reports concerning the direct inhibition of ROS by cannabinoids use different cells and stimuli for ROS generation, including lipopolysaccharide (LPS), UV radiation and hydrogen peroxide [23,26,27,28]. In fact, the inhibition of ROS by CBD in keratinocytes has been described as indirect, due to the blockage of the nuclear factor erythroid 2-related factor 2 (Nrf2) signaling pathway, which regulates ROS production through the reduction in the activity of ROS-generating enzymes [28,29,30]. However, previous findings have shown that HaCaT cells exposed to urban particulate matter show an upregulation of nuclear factor kappa-B (NF-κB) and cyclooxygenase (COX)-2 [31]. It has also been shown that CBD may interfere with Nrf2 biological functions in a concentration-dependent manner, affecting the Nrf2 and Nf-κB pathways [32]. For example, a report by Jastrząb, Gęgotek and Skrzydlewska [30] demonstrated that CBD at 1 μM upregulated the Nrf2 pathway while downregulating the Nf-κB pathway in UV-irradiated keratinocytes. Thus, the lack of inhibition observed in our results may be a direct result of the concentration used. In our pollution-induced inflammation model, CBD exerted anti-inflammatory activity by significantly reducing the levels of the pro-inflammatory cytokines IL-6 and IL-1α, which is in accordance with multiple reports [16,19,33].

Regarding CBG, it significantly decreased IL-1α levels while not impairing IL-6 amounts. Data indicating a similar trend have been reported for HaCaT cells exposed to both commensal and pathogenic bacteria [10]. Although there are fewer reports on the biological properties of CBG than CBD, multiple studies have demonstrated the differences between the two cannabinoids, as they seem to react differently to the same cell receptors, justifying their differences in anti-inflammatory activity [34,35]. This has been shown for cannabinoid (CB) receptors 1 and 2, which are involved in several inflammation processes, and while CBG acts as a weak or partial agonist at CB_1_ and CB_2_, CBD acts as an antagonist of the same receptors [35,36].

As a second objective, we studied the skincare potential of CBD and CBG, both of which demonstrated the capacity to inhibit skin aging enzymes *in chemico*. CBD inhibited MMP-1’s activity by more than 60%, indicating its potential to prevent collagen degradation in the ECM. This finding aligns with a study by Gęgotek, et al. [37], where CBD significantly reduced MMP-1 expression in UVB-treated keratinocytes. Moreover, our results showed that CBG could inhibit MMP-1, elastase, and tyrosinase. This is consistent with a report by Gaweł-Bęben, et al. [38], which demonstrated that CBG significantly inhibited mushroom tyrosinase activity. However, CBD did not reduce the activity of either mushroom or murine tyrosinase. To the best of our knowledge, this is the first report of the potential inhibitory activity of CBG regarding MMP-1 and elastase. Next, we assessed the impact of CBD and CBG on ECM proteins in skin-derived cultured cells and skin explants to validate their anti-aging potential. Collagen and fibronectin are relevant in anti-aging studies, as they are two key proteins of the ECM that contribute to dermal stability, preventing wrinkling [39,40]. Our in vitro and ex vivo experimental models showed that neither CBD nor CBG were able to increase the levels of these proteins or those of other components tested. This contrasts with previous studies where CBD increased the expression levels of collagen and hyaluronic acid, although it did not promote fibronectin production [24,41]. However, in both studies, the expression of protein levels after exposure to cannabinoids was assessed in stress-induced models, such as an in vitro cell model of stress-induced premature senescence and an in vitro cell model of UV-radiation exposure. The scarcity of literature concerning the anti-aging effects of CBD and CBG, along with the results obtained here, suggests that these molecules may be unable to increase ECM protein levels, not preventing wrinkling and dermal losses under healthy basal conditions. Conversely, we hypothesize that the observed anti-inflammatory activity in response to air-borne pollution may contribute to restoring these environments and normal skin markers. Thus, these cannabinoids may still be valuable as ingredients in cosmetic products as they both demonstrated the inhibition of skin aging enzymes, a protective effect against inflammation caused by urban air pollution, and high skin permeation values. Both CBD and CBG had an absorbed dose of approximately 70%, resulting in an average absorbed dose between 510 and 520 μg/cm^2^ for both cannabinoids at 0.5% (*w*/*v*). In vitro permeation studies with CBD and CBG yielded lower absorbed doses compared to those obtained here. A recent study demonstrated an absorbed dose of 23 μg/cm^2^ and 242.41 μg/cm^2^ for 1% and 10% (*w*/*v*) CBD solutions, respectively. In another study using gel enhancers and a 10% (*w*/*w*) CBD or CBG solution, results showed 198.40 μg/cm^2^ and 136.06 μg/cm^2^, respectively, after a 24 h period [42,43]. It is probable that the combined use of sunflower oil and squalane contributed to increased skin permeation, as both have been described as excellent permeation enhancers [44,45,46]. This allowed for a higher absorbed dose, even with a lower concentration than those reported elsewhere. Moreover, considering the available literature, we propose that the anti-inflammatory effect demonstrated by CBD and CBG may help restore epidermal balance and, consequently, upregulate ECM proteins, potentially preventing aging [47,48,49]. Nonetheless, further research is needed to solidify these findings and unravel the molecular mechanism underlying the function of CBG and CBD as soothing agents.

## 4. Materials and Methods

### 4.1. Cannabinoids’ Preparation

For the assays performed, cannabidiol (CBD) and cannabigerol (CBG) were used. A CBD isolate (CBD), purified from hemp seeds (purity ≥ 98%), was purchased from Mile High Labs (Lot: IL2004R007B; Loveland, CO, USA), and a CBG isolate (purity ≥ 98%) was obtained via fermentation by Amyris (Lot: 9194; Emeryville, CA, USA). For the in vitro assays, CBD and CBG were dissolved in ethanol (Sigma-Aldrich, Steinheim, Germany) at a concentration of 60% (*v*/*v*). For the assays involving ex vivo models, all compounds were dissolved at a concentration of 0.5% (*w*/*v*) using sunflower oil (Sigma-Aldrich, Steinheim, Germany) and squalane (Amyris, Emeryville, CA, USA), at a 1:4 ratio.

### 4.2. Cell Culture Conditions

HaCaT, a keratinocyte cell line, was obtained from Cytion (300493, Eppelheim, Germany) and used within passages 35 to 45. Human dermal fibroblasts (HDFa; primary cell line, adult) were obtained from American Type Culture Collection (ATCC; PCS-201-012™, Manassas, VA, USA) and used within passages 4 to 8. Unless otherwise specified, cells were cultured using high glucose Dulbecco’s Modified Eagle’s Medium (DMEM; Gibco, Paisley, Scotland) supplemented with 10% (*v*/*v*) heat-inactivated Fetal Bovine Serum (FBS; Gibco, Paisley, Scotland) and 1% (*v*/*v*) penicillin-streptomycin (Pen-Strep; Gibco, Paisley, Scotland).

THP-1 cells were obtained from ATCC (TIB-202™, Manassas, VA, USA) and were cultured in RPMI 1640 (Gibco, Paisley, Scotland), supplemented with 10% (*v*/*v*) FBS, 0.05 mM 2-mercaptoethanol (Gibco, Paisley, Scotland), and 1% (*v*/*v*) Pen-Strep. TK6 cells, also obtained from ATCC (CRL-8015™; Manassas, VA, USA), were cultured in RPMI 1640, supplemented with 10% (*v*/*v*) FBS and 1% (*v*/*v*) Pen-Strep.

All cells were incubated at 37 °C in a humidified atmosphere with 5% CO_2_.

### 4.3. Cell Viability Assay

For adherent cells, namely HaCaT and HDFa, cell viability was examined with the PrestoBlue™ Cell Viability Reagent (Thermo Scientific, Loughborough, UK) according to the manufacturer’s instructions. Briefly, cells were seeded at a density of 1 × 10^5^ cell/mL in 96-well plates and incubated overnight. The culture media was then replaced with fresh culture media containing different concentrations of CBD or CBG. After 24 h of incubation, PrestoBlue™ reagent was added, and the fluorescence was measured after 1 h of incubation, at 545 nm excitation and 590 nm emission using a Synergy HT plate reader (BioTek Instruments, Winooski, VT, USA). Dimethyl sulfoxide (DMSO; Thermo Scientific, Loughborough, UK) at a 10% (*v*/*v*) concentration was used as a positive control for cell death, and fresh culture media was used as a positive control for cell viability. All assays were performed in triplicate, with four replicates each. A cytotoxic effect was considered when metabolic inhibition exceeded 30%, following the guidelines outlined in ISO 10993-5 [50].

Regarding the non-adherent cells TK6, cell viability was assessed by flow cytometry with propidium iodide (PI). Briefly, cells were collected by centrifugation (1500 rpm, 5 min), and a cell suspension with a concentration of 2 × 10^6^ cells/mL was prepared. Afterward, cells were mixed with the test cannabinoids at various concentrations and incubated for 24 h. The experimental controls were identical to the ones used for the viability assay with the adherent cells. Following incubation, cells were centrifuged and washed twice with phosphate-buffered saline (PBS) containing 0.1% (*w*/*v*) bovine serum albumin (BSA, Thermo Scientific, Loughborough, UK). Then, cells were resuspended in 200 μL of PBS-0.1% BSA, after which 10 μL of PI solution at a concentration of 12.5 μg/mL was added. After a 10 min incubation in the dark, samples were analyzed using an Accuri C6 Plus flow cytometer (BD Biosciences, Franklin Lakes, NJ, USA), with the BD Accuri™ C6 Plus Software version 1.0.27.1 (BD Biosciences, Franklin Lakes, NJ, USA).

### 4.4. Skin Sensitization

#### 4.4.1. *In Chemico*: Direct Peptide Reactivity Assay (DPRA)

The Direct Reactivity Peptide Assay (DPRA) was performed following the guidelines outlined in the Organization for Economic Co-operation and Development (OECD) test guideline No. 442C [51]. Test chemicals were prepared by diluting CBD and CBG in 60% (*v*/*v*) ethanol to attain two concentrations, 25 and 5 μM. The samples were homogenized at 40 °C for 30 min and then sonicated using a sonication bath for 15 min. Cysteine or lysine peptides were subsequently mixed with the samples at a 1:10 or 1:50 ratio, respectively. Peptide depletion was assessed after 24 h using reverse-phase high-performance liquid chromatography (HPLC; Agilent 1260 Infinity II) equipped with a Zorbax C18 column. Detection was carried out at 220 nm using an Agilent 1260 Diode Array Detector (DAD HS), using an external standard calibration curve for each peptide. The percentage of peptide depletion was calculated for each peptide. Peptide depletion percentage was calculated for each peptide. Samples exhibiting an average depletion of both cysteine and lysine combined above 6.38%, or showing cysteine depletion alone above 13.89%, were considered as having sensitizing potential. Cinnamic aldehyde was used as a positive control.

#### 4.4.2. In Vitro: Human Cell Line Activation Test (h-CLAT)

1. Cytotoxicity test and determination of CV75

The evaluation of in vitro skin sensitization potential was performed in accordance with the guidelines outlined in OECD test guideline No. 442E [52]. Briefly, THP-1 cells were seeded at a concentration of 1.0 × 10^6^ cells/mL in 24-well plates with various concentrations of each test cannabinoid and incubated overnight. After washing twice with PBS-0.1% BSA (FACS buffer, Thermo Scientific, Loughborough, UK), the cells were stained with PI at a concentration of 0.625 μg/mL (Sigma-Aldrich, Steinheim, Germany) and analyzed using flow cytometry. A 10% (*v*/*v*) DMSO solution was used as a positive control for cell death. Cell viability was defined as the ratio of living cells (PI negative cells) to the total number of acquired cells. The test concentration that resulted in a cell viability of 75% (CV75) was calculated using log-linear interpolation, as shown in the equation below:

(1) $$Log\;CV75=\frac{\left(75-c\right)\times Log\;b-\left(75-a\right)\times Log\;d}{a-c}$$

where a and c correspond to the closest cell viability percentage to 75%, and b and d correspond to the sample’s concentration. A total of 8 doses based on the CV75 were tested.

2. h-CLAT Assay

After determining the CV75, THP-1 cells (1.0 × 10^6^ cells/mL) were exposed to each cannabinoid at eight different doses. After a 24 h exposure, cells were washed twice with FACS buffer and incubated with Human TruStain FcX™ (BioLegend, San Diego, CA, USA) for 20 min at RT. Next, cells were stained for 30 min at 4 °C using the following fluorescein isothiocyanate (FITC)-conjugated monoclonal antibodies (mAbs): anti-human CD54 (1:10; 353108), anti-human CD86 (1:10; 374204) and FITC labeled-mouse IgG1, k Isotype Ctrl (1:25; 981802), all from BioLegend (San Diego, CA, USA). After thorough washing and suspension in FACS buffer, the fluorescence intensity for a total of 10.000 PI-negative living cells was measured by flow cytometry. To assess CD54/CD86 expression, cell viability and FITC-mean fluorescence intensity (MFI) values were collected, and the relative fluorescence intensities (RFI54 and RFI86) were calculated according to the following formula:

(2) $$RFI=\frac{MFI\;of\;chemical\;treated\;cells-MFI\;of\;chemical\;treated\;isotype\;cells}{MFI\;of\;solvent\;treated\;cells-MFI\;of\;solvent\;treated\;isotype\;cells}\times 100$$

Any given agent is classified as a sensitizer whenever CD86 and/or CD54 RFI values are above the positive criteria (CD86 ≥ 150 and CD54 ≥ 200). Nickel(II) Sulphate (Sigma-Aldrich, Steinheim, Germany) at 10 mg/mL was used as a sensitization control.

### 4.5. Genotoxicity

#### 4.5.1. Micronucleus

To assess the impact of cannabinoids on chromosome structure, micronucleus analysis was performed using the MicroFlow^®^ kit (BD Biosciences, Franklin Lakes, NJ, USA), according to the manufacturer’s instructions and in accordance with OECD guideline Test No. 487: In Vitro Mammalian Cell Micronucleus Test [53]. Initially, the cytotoxicity of cannabinoids against TK6 cells was assessed. Afterward, cells were incubated with 50, 25 or 2.5 μM of each test cannabinoid and cultured in 24-well plates (1.0 × 10^6^ cells/mL) for 24 h. Mitomycin C from *Streptomyces caespitosus* at 25 ng/mL (Sigma-Aldrich, Steinheim, Germany) was used as a positive control for micronuclei formation. The generation of micronuclei was assessed by flow cytometry. The assay was repeated twice, and each condition was tested in triplicate.

#### 4.5.2. AMES Fluctuation Test

To assess genotoxic potential, the Ames MOLTOX^®^ FT™ 471 test was performed in accordance with the manufacturer’s instructions and with OECD guideline Test No. 471, Bacterial Reverse Mutation Test [54]. In brief, the ability of cannabinoids to induce reversion of histidine-requiring *Salmonella typhimurium* strains TA98, TA100 and TA102 was evaluated. These strains were exposed to a range of concentrations (125, 100, 75, 50 and 25 μM) of each cannabinoid for 90 min. Subsequently, cultures were diluted in a histidine-free medium and incubated for two days. Histidine + revertants were identified by a change in medium color change (from purple to yellow) and counted. A significant increase was defined as a ≥2-fold increase in revertant numbers when compared to the baseline control. Moreover, this assay was performed with and without metabolic activation, using Phenobarbital/β-Naphthoflavone induced Sprague Dawley rat liver S9. The assay was performed twice, with each condition tested in triplicate.

### 4.6. In Vivo Safety Assays

To assess the safety of cannabinoids for topical use, four contact tests were conducted: primary irritability, cumulative dermal irritability, dermal phototoxicity, and photosensitization. The study was a single-blind, controlled, single-center clinical trial. The test product and control were spread on filter paper discs (patches, 50 μL/cm^2^), applied on the upper back of the research participants, and covered with semi-occlusive hypoallergenic tape. CBD and CBG were used at 0.05% (*w*/*v*). Saline solution was used as the control.

The research participants were evaluated by a dermatologist at the beginning and end of the study and followed throughout the course of the study. A total of 65 research participants were included. However, only 58 research participants completed the study, due to personal reasons unrelated to the study. Participants were male and female, considered healthy, and aged between 18 and 70 years old. This trial was performed by KosmoScience Ciência & Tecnologia Cosmética Ltda (Valinhos, São Paulo, Brazil), with the respective report being issued on 31 October 2023.

#### 4.6.1. Primary Irritability Potential, Accumulated Dermal Irritability and Dermal Sensitization

For the evaluation of primary irritability, the area was evaluated after 48 and 96 h of the first application of the cannabinoids. For the evaluation of accumulated dermal irritability, every 48 h, the research participants returned to have the patch removed, with the area of analysis being cleaned and analyzed after 30 min of rest. This procedure was repeated over a period of three weeks. The applications were performed three times a week for three consecutive weeks, on alternate days, making a total of nine applications. After the described procedures, there was a period of at least 10 days of rest, when no patches were applied to the back of the research participants (rest phase). After the rest phase, cannabinoids were applied again for the sensitization assessment. After the removal of the samples, the area was evaluated after 48 and 72 h. Conditions related to the classification of skin irritation potential are described in Supplementary Tables S2 and S3.

#### 4.6.2. Dermal Phototoxicity and Dermal Photosensitization

The application of the patch was performed as described in Section 4.6.1. After approximately 24 h of each application, the patch was removed, and after waiting 30 min, the area received ultraviolet irradiation (UVA + UVB) for the assessment of phototoxicity. Readings were taken after each irradiation and no patch was applied to the back of the research participants (rest phase) for at least 10 days. After the rest phase, the test product was applied once again to a new area to assess photosensitization, remaining in contact with the skin for a period of 24 h (challenge phase). After removing the patch and waiting 30 min, one of the areas received ultraviolet radiation. The irradiation was only performed in one of the applied areas, while the other was spared from irradiation and used as a control. The area was evaluated right after irradiation and after 24, 48 and 72 h. The conditions related to ultraviolet irradiation are described in Supplementary Table S1.

### 4.7. Antioxidant Assays

#### 4.7.1. β-Carotene/Linoleic Acid Assay

The antioxidant capacity of cannabinoids was evaluated using a β-carotene/linoleic acid system according to the modified literature procedure [55]. Briefly, a stock solution of β-carotene/linoleic acid was prepared by dissolving 20 mg of β-carotene (Sigma-Aldrich, Steinheim, Germany) into 1 mL of chloroform (HPLC grade, Sigma-Aldrich, Steinheim, Germany). Subsequently, a mixture of 40 μL of linoleic acid and 530 μL of Tween 40 (Merck, Darmstadt, Germany) was prepared, and then 50 μL of the previously prepared β-carotene was added. Chloroform was evaporated under nitrogen, and distilled water was added with vigorous shaking until absorbance reached 0.7 (±0.02) at 485 nm. Then, 15 μL of each test cannabinoid were added to 185 μL of the β-carotene/linoleic acid solution and incubated at 45 °C. The absorbance of the mixtures was measured immediately at 485 nm (t0), and at 15 min intervals for 120 min, using a Synergy HT plate reader (BioTek Instruments, Winooski, VT, USA). Butylated hydroxytoluene (BHT; Sigma-Aldrich, Steinheim, Germany) was used as a positive control, and a standard curve was prepared using successive dilutions (T0 = 0.15 mg/mL). Ethanol was used as a blank. Antioxidant capacity of the samples was compared with those of BHT and expressed as percentage of inhibition in relation to the antioxidant control, as seen in the equation below:

(3) $$\%\;\mathrm{Inhibition}=\frac{{\left[A_{0}-A_{1}\right]}_{Ct}}{{\left[A_{0}-A_{1}\right]}_{Sample}}\times 100$$

where *A*_0_ is the absorbance at time 0, and *A*_1_ is the absorbance after the 120 min reaction of the β-carotene/linoleic acid solution with the sample.

#### 4.7.2. 2,2-diphenyl-1-picrylhydrazyl (DPPH) Radical Scavenging Assay

To assess the antioxidant capacity of cannabinoids, a modified 2,2-diphenyl-1-picrylhydrazyl (DPPH) radical scavenging assay was performed [56]. Briefly, CBD and CBG were dissolved in ethanol at 1 mg/mL, and successive dilutions were prepared (0.5 mg/mL to 0.0079). Then, 25 μL of the sample was added to 175 μL per well of an adjusted (0.600 ± 0.100 at 515 nm) DPPH solution. A standard curve with 6-hydroxy-2,5,7,8-tetramethylchroman-2-carboxylic acid (Trolox; Sigma-Aldrich, Steinheim, Germany) was generated and used to determine a positive control (0.005–0.08 mg/mL). The mixture of samples and DPPH was then incubated in the dark for 30 min at RT. The absorbance was measured at 515 nm using a Synergy HT plate reader (BioTek Instruments, Winooski, VT, USA). The assay was performed three times, and each condition was tested in triplicate. The results were expressed in μM of Trolox equivalents (TE)/100 g DM. The radical scavenging activity (RSA) was calculated using the equation:

%RSA = [(A_DPPH_ − A_S_)/A_DPPH_] × 100 (4)

where A_S_ is the absorbance of the solution with the sample and A_DPPH_ is the absorbance of the DPPH solution. The concentrations responsible for 50% of RSA (IC50 values) were calculated from the graphs of RSA percentages plotted against the concentration of cannabinoids.

#### 4.7.3. Exposure to Urban Particulate Matter

##### Reactive Oxygen Species (ROS) Assay

The potential of cannabinoids to prevent the generation of reactive oxygen species (ROS) was assessed using the 2′,7′-dichlorofluorescein diacetate (DCFDA; Sigma-Aldrich, Steinheim, Germany) probe. Briefly, HaCaT cells were seeded at a 1 × 105 cells/mL concentration in 96-well plates in medium without phenol red. After 24 h, cells were exposed to Urban Particulate Matter SRM1648a (UPM, National Institute of Standards and Technology, Gaithersburg, MD, USA) at 500 μg/mL. Phorbol-12-Myristate-13-Acetate (PMA; Sigma-Aldrich, Steinheim, Germany) at 125 nM and ascorbic acid (Sigma-Aldrich, Steinheim, Germany) at 5 mM were used as a positive control for ROS generation and as a control for ROS inhibition, respectively. Plates were then incubated for 24 h. A DCFDA probe was diluted and added to each well at a final concentration of 25 μM. Fluorescence at Ex/Em = 495/529 nm was immediately read at 1 h intervals for 4 to 6 h.

##### Quantification of Cytokines

HaCaT cells were seeded at 2.5 × 10^5^ cells/mL in 12-well plates. After 24 h, cells were exposed to 500 μg/mL UPM SRM1648a, with or without cannabinoids at 10 μM. Betamethasone (Sigma-Aldrich, Steinheim, Germany) at 20 μM was used as a positive control. After a 24 h exposure, supernatants were collected, centrifuged (10,000 rpm, 10 min), and IL-1α and IL-6 concentrations were determined using an enzyme-linked immunosorbent assay (ELISA) (BioLegend, San Diego, CA, USA), according to the manufacturer’s instructions. Additionally, total protein content was extracted using a lysis buffer (50 mM Tris-HCl, pH 7.8, 150 mM NaCl, 1 mM EGTA, 1.5 Mm MgCl_2_, 0.4% (*w*/*v*) sodium dodecyl sulfate (SDS, Merck, Darmstadt, Germany), 1 μL/mL benzonaze (25 U/mL), 1% (*v*/*v*) Nonidet-P40, and protease inhibitor cocktail tablets (Roche, Basel, Switzerland)), quantified with the Pierce™ BCA Protein Assay Kit (Thermo Scientific, Loughborough, UK) and used to normalize ELISA’s results. Two independent experiments were performed, and each condition was tested in triplicate.

### 4.8. Skincare Properties: In Vitro Evaluation

#### 4.8.1. Inhibition of Skin-Related Enzyme Activity

To study the skin-care potential of cannabinoids, we evaluated their impact on the activity of different skin-aging enzymes, namely elastase, matrix metalloproteinase-1 (MMP-1) and tyrosinase. To do so, we used the neutrophil elastase inhibitory screening kit (fluorimetric) (ab118971, abcam, Cambridge, UK), the matrix metalloproteinase-1 (MMP-1) inhibitor screening kit (ab139443, abcam, Cambridge, UK), and the tyrosinase inhibition colorimetric screening kit (ab204715, abcam, Cambridge, UK), respectively, according to the manufacturer’s instructions. Cannabinoids were dissolved in 60% (*w*/*v*) ethanol to the final concentrations of 100, 50, 10 and 5 μM, which were tested. In all assays, an enzyme control (enzymatic activity without any interference) was made with a mixture of enzyme and substrate in the presence of a buffer. The absorbance of each well was measured using a Synergy HT plate reader (BioTek Instruments, Winooski, VT, USA). To establish the inhibition values, data were plotted as OD versus time for each sample, and the range of time points during which the reactions were linear was selected to calculate enzyme inhibition activity.

#### 4.8.2. Quantification of Skin Markers

The levels of human pro-collagen 1α1 and fibronectin were measured in HDFa, and human cytokeratin 14 levels were assessed in HaCaT using cell culture supernatant and cell extract samples with abcam SimpleStep ELISA kit™ (ab229389, ab229398 and ab226895, respectively; Cambridge, UK). After seeding (2.5 × 10^5^ cells/mL) in 12-well plates, cells were exposed to CBD and CBG at concentrations of 10 and 5 μM for 24 h. Afterward, cells’ supernatants were collected and centrifuged, while protein extraction was performed using the extraction buffer provided by the kits. The assays were performed according to the manufacturer’s instructions. The concentration of the target proteins was determined by interpolating the fluorescence values with the blank control subtracted against the standard curve for each protein. Palmitoyl Tripeptide-1 (0.5 μM, Pal-GHK; Cayman Chemical, Ann Harbour, MI, USA) was used as a reference. All assays were performed in triplicate.

#### 4.8.3. Western Blot

Collagen 1 and fibronectin protein production was evaluated by Western Blot. After seeding (2.5 × 10^5^ cells/mL) in 12-well plates, cells were exposed to CBD and CBG at concentrations of 10 and 5 μM for 24 h. Following exposure, the cells were lysed in lysis buffer, and the protein content was quantified using the Pierce™ BCA protein assay (Thermo Scientific, Loughborough, UK). The proteins were then subjected to sodium dodecyl-sulfate polyacrylamide gel electrophoresis (SDS-PAGE) and transferred onto a PVDF membrane (Immobilon-P, Millipore; Merck, Darmstadt, Germany). The membranes were blocked with Tris-buffered saline (TBS) containing 0.1% (*v*/*v*) Tween-20 (Merck, Darmstadt, Germany) and 2% (*w*/*v*) BSA (Thermo Scientific, Loughborough, UK) for 1 h. Afterward, the membranes were incubated overnight in blocking solution containing anti-collagen I (1:100, ab34710, abcam, Cambridge, UK) or anti-fibronectin primary antibody (1:100, ab2413, abcam, Cambridge, UK) and washed three times with TBS containing 0.1% Tween-20. Subsequently, the membranes were incubated with horseradish (HRP)-conjugated goat anti-rabbit IgG secondary antibody (ab6721, abcam, Cambridge, UK), triple-washed in TBS buffer, and developed using the ECL™ Prime Western Blotting system (Cytiva, Marlborough, MA, USA). Images were captured as .tiff files and processed using ImageJ software (v1.53o, updated 11 January 2022).

### 4.9. Anti-Aging

#### 4.9.1. Skin Explants

Samples were harvested from routine abdominoplasties of healthy anonymous donors, aged 32 to 45 years. Written informed consent from all participants was obtained under the protocol established and approved by the Ethical Committee of Hospital Lusíadas (Porto, Portugal) and Universidade Católica Portuguesa (UCP, Porto, Portugal). Before any experiment, the fat adipose tissue was carefully removed with scissors, taking care to preserve the dermis from damage, and 12 mm-diameter or 20 mm-diameter punch biopsies were excised for anti-aging and skin permeation studies, respectively. For the anti-aging studies, 25 μL from each formulation was topically applied once daily, for three days. An oil preparation without cannabinoids was used as a vehicle control. Matrixyl 3000 (3% (*v*/*v*), LotionCrafter, Eastsound, WA, USA) was used as a positive control for ECM production in the anti-aging assays.

#### 4.9.2. Gene Expression Analysis

##### RNA Extraction and cDNA Conversion

Cells were seeded at a density of 2.5 × 10^5^ cells/mL, and after 24 h, culture media was replaced with fresh media containing each test cannabinoid at concentrations of 5 and 10 μM. After a 24 h exposure, RNA was extracted from cells using the Qiagen RNeasy extraction kit (Hilden, Germany), according to the manufacturer’s instructions. To extract RNA from tissues, a small tissue sample was excised from each punch biopsy (max 30 mg) prior to fixation. Samples were then incubated overnight at 55 °C in digestion buffer with 100 μg/mL of proteinase K (PK; NZYTech, Lisbon, Portugal) and 0.5% (*w*/*v*) SDS (Merck, Darmstadt, Germany). Afterward, samples were incubated at 80 °C for 15 min to completely inactivate PK. Total RNA was quantified by spectrophotometry using ScanDrop (Analytik Jena, Jena, Germany). RNA was converted to cDNA using the NZY First-Strand cDNA Synthesis Kit (NZYTech, Lisbon, Portugal), according to the manufacturer’s instructions. Two independent experiments were performed, and each condition was tested in triplicate.

##### qPCR

Real-time quantitative polymerase chain reaction (qPCR) was used to quantify the relative expression levels of target genes. NZYSupreme qPCR Green Master Mix (NZYTech, Lisbon, Portugal) was used to amplify the genes with 10 ng cDNA per well in combination with 1 μM of respective Forward/Reverse primer set (Supplementary Table S4). qPCR reactions were prepared to a final volume of 10 μL, containing 1× NZYSupreme qPCR Green Master Mix (NZYTech, Lisbon, Portugal), 0.5 to 1 μM of forward and reverse primers (Integrated DNA Technologies, Coralville, IA, USA), 1 μL of DNA-Free Water (Qiagen, Hilden, Germany) and 2 μL of cDNA. The qPCR was performed in a qTOWER^3^ G (Analytik Jena, Jena, Germany) touch Real-time system (Analytik Jena, Jena, Germany) coupled with the Analytik Jena qPCRsoft 4.0 program. Cycle conditions were as follows: 10 min at 95 °C, followed by 40 cycles of denaturation at 95 °C for 15 s and annealing/extension at 60 °C for 1 min. The amplification steps were followed by a melt dissociation step to check for nonspecific product formation. Additionally, a non-template control (NTC) using sterile water was also included for all primers tested to exclude any external nucleic acid contamination. Three independent experiments were performed, and each condition was tested in triplicate.

#### 4.9.3. Histological Analysis

At day 3 for skin, samples were fixed in 10% (*v*/*v*) neutral buffered formalin (Bio-Optica, Milan, Italy), dehydrated, embedded in paraffin using a tissue processor (Leica Biosystems, Nussloch, Germany) and cut into 5 μm sections (Microtome CUT 5062, SLEE medical GmbH, Nieder-Olm, Germany). Tissue sections were stained with Masson’s trichrome stain (Bio-Optica, Milan, Italy) following routine protocols. Samples were examined using an upright Imager.M1 Microscope, and image processing was performed using Zen Software 3.2.

#### 4.9.4. Image Analysis

To infer the collagen amount, five images of random fields were acquired for each condition and independent experiment. The images were automatically quantified with the Cell Profiler software (v 4.2.5) using an algorithm for the identification of blue pixels (collagen) keeping the same color segmentation settings for each image. The amount of collagen was calculated as a percentage of total pixels.

#### 4.9.5. In Vitro Skin Permeation Study

To study the skin permeation of CBD and CBG, an in vitro study was performed in accordance with the OECD test guideline No. 428 “Skin absorption—in vitro method” [57], under occlusive conditions using modified Franz diffusion cells (permeation area: 0.636 cm^2^; receptor chamber volume: 5 mL) and the human skin explant as a membrane. Briefly, before the experiments, skin explants were thawed at RT and placed in each cell. At the beginning of the permeation experiment, 0.1 mL of each formulation containing 0.5% (*w*/*v*) of cannabinoids was applied in duplicate, onto the *Stratum corneum* of each epidermis sample in the donor compartment of each cell. Receptor compartments were filled with 60% (*v*/*v*) ethanol solution. The gap between the upper and lower parts of the Franz cells was fastened together using a clamp. The system was kept at 32 °C by a circulating water bath and the membrane surface temperature was also kept at 32 °C throughout the 24 h of the experiment. After 24 h, the skin was removed from the Franz diffusion cell and each side was gently treated with 1 mL of 60% (*v*/*v*) ethanol to wash out the unabsorbed cannabinoids. Then, the membrane was minced by ultra-turrax (IKA, Staufen, Germany) with 4 mL of 60% (*v*/*v*) ethanol. The resulting solution was centrifuged (8000 rpm, 5 min) and the supernatant, the unabsorbed dose solution and the receptor cell solution were filtered (0.45 μm, Millipore) for further analysis.

##### Chromatographic Conditions

The quantification of cannabinoids was performed by liquid chromatography–electrospray ionization–ultrahigh-resolution–quadrupole time of flight–mass spectrometry (LC-ESI-UHR-QqTOF-MS), by external calibration of CBD and CBG standards. All samples retrieved from the Franz cells were diluted in a solution of 60% (*v*/*v*) ethanol and filtered before injection. The chromatographic separation was carried out with a Bruker Elute series equipped with a UHR-QqTOF mass spectrometer (Impact II, Bruker Daltonics, Billerica, MA, USA) and a BRHSC18022100 intensity Solo 2 C18 column (100 × 2.1 mm, 2.2 μm, Bruker Daltonics, Billerica, MA, USA). Binary A/B gradient (solvent A: Milli Q water with 0.1% formic acid; solvent B: acetonitrile with 0.1% formic acid) was established as follows: initial conditions were 50% B, raised to 100% B over 4 min, held at 100% B until 6 min, decreased to 50% B over the next 6 min, with a total running time of 12 min. A flow rate of 0.25 mL/min was used. The column temperature was set to 37 °C, and the injection volume was 5 μL.

The MS acquisition was set to positive ionization mode with the selected parameters: end plate offset voltage: 500 V; capillary voltage: 3.0 kV; drying gas temperature: 200 °C; drying gas flow: 8.0 L/min; nebulizing gas pressure: 2 bar; collision radio frequency (RF): from 250 to 1000 Vpp; transfer time: from 21.4 to 60 μs; collision cell energy: 10 eV.

### 4.10. Statistical Analysis

Statistical analysis was performed using the IBM SPSS Statistics v21.0.0 (USA) software. Normality of the distributions was evaluated using Shapiro–Wilk’s test. For the data that followed a normal distribution and where the homoscedasticity of variances was verified, one-way analysis of variance (ANOVA) coupled with Tukey’s post hoc test was used to assess the differences between the results. Statistical significance was considered for *p*-values below 0.05. For data not exhibiting a normal distribution, the mean comparison between independent samples was performed using the Mann–Whitney’s test.

## 5. Conclusions

The cosmetic market is in constant exponential growth and in search of new bioactive ingredients to produce cosmeceutical formulations. Cannabinoids do not seem capable of exerting the “standard” anti-aging effects, as they show little to no effect in the expression of ECM-related skin-care biomarkers and prevent epidermal wrinkling. However, we show that they are safe for topical application and may successfully reduce inflammation and oxidative stress, albeit indirectly. The combination of CBD and CBG with known anti-aging molecules could present a possibility to circumvent the lack of anti-aging action while adding the anti-inflammatory and protective activity of these cannabinoids. Still, further studies are necessary to support our preliminary results and to explore the underlying molecular mechanisms. Combined with its known anti-inflammatory properties, cannabinoids could be a potential ingredient as a soothing agent and used for skin inflammation treatment.

## Figures and Tables

**Table 1:** Results for *in chemico* skin sensitization through the DPRA, as described by OECD guideline 442C. Any given agent is classified as a sensitizer whenever samples exhibit an average depletion of both cysteine and lysine combined above 6.38% or cysteine depletion alone above 13.89%.

	Mean Peptide Depletion (%)					Potential Sensitizer?		
	Cysteine	Lysine	Mean of Cysteine and Lysine % Depletion	Reactivity (Cysteine Only)	Reactivity (Cys and Lys)	Based on Cysteine Only Prediction Model	Based on Mean of Cysteine and Lysine Prediction Model	Potential Sensitizer?
CBD 25	32.2	0.00	16.1	Moderate	Low	Positive	Positive	Yes
CBD 5	6.90	0.70	3.80	Minimal	Minimal	Negative	Negative	No
CBG 25	7.20	2.70	4.90	Minimal	Negative	Negative	Negative	No
CBG 5	5.90	2.70	4.30	Minimal	Negative	Negative	Negative	No
Positive CTR	70.9	59.5	65.2	Moderate	High	Positive	Positive	Yes

**Table 2:** Results for the hCLAT assay, as described by OECD guideline 442E. Any given agent is classified as a sensitizer whenever CD86 and/or CD54 RFI values are above the positive criteria (CD54 ≥ 200 and CD86 ≥ 150).

			RFI		Sensitizer?
		Cell Viability (%)	CD54	CD86	
Negative CTR		98%	100	100	-
Positive CTR		87%	284	294	Yes
CBG	51.1 μM	80%	144	75.1	No
	42.5 μM	85%	133	79.5	No
CBD	45.6 μM	87%	113	112	No
	38.6 μM	87%	76.1	132	No

**Table 3:** AMES test results for CBG using *Salmonella typhimurium* strains TA98, TA100 and TA102, with and without metabolic activation (S9). In accordance with the kit used, a significant increase occurs when there is a ≥2-fold increase in revertant numbers.

	**TA98 Without S9**		**TA98 with S9**	
	**Fold Increase**	**Significant?**	**Fold Increase**	**Significant?**
Baseline	1.00	No	1.00	No
Pos CTR	2.87	Yes	24.6	Yes
125 μM	0.35	No	1.04	No
100 μM	0.12	No	1.05	No
75 μM	0.24	No	0.87	No
50 μM	0.47	No	0.52	No
25 μM	0.43	No	0.17	No
	**TA100 Without S9**		**TA100 with S9**	
	**Fold Increase**	**Significant?**	**Fold Increase**	**Significant?**
Baseline	1.00	No	1.00	No
Pos CTR	2.01	Yes	4.76	Yes
125 μM	0.29	No	1.15	No
100 μM	0.22	No	0.97	No
75 μM	0.48	No	0.57	No
50 μM	0.41	No	1.10	No
25 μM	0.25	No	0.97	No
	**TA102 Without S9**		**TA100 with S9**	
	**Fold Increase**	**Significant?**	**Fold Increase**	**Significant?**
Baseline	1.00	No	1.00	No
Pos CTR	2.00	Yes	2.01	Yes
125 μM	0.88	No	1.04	No
100 μM	0.87	No	1.00	No
75 μM	0.81	No	1.01	No
50 μM	1.03	No	1.09	No
25 μM	1.01	No	1.07	No

**Table 4:** AMES test results for CBD using *Salmonella typhimurium* strains TA98, TA100 and TA102, with and without metabolic activation (S9). In accordance with the kit used, a significant increase occurs when there is a ≥2-fold increase in revertant numbers.

	**TA98 Without S9**		**TA98 with S9**	
	**Fold Increase**	**Significant?**	**Fold Increase**	**Significant?**
Baseline	1.00	No	1.00	No
Pos CTR	2.87	Yes	24.6	Yes
125 μM	0.59	No	1.39	No
100 μM	0.71	No	0.69	No
75 μM	0.35	No	0.51	No
50 μM	0.10	No	0.57	No
25 μM	0.34	No	0.70	No
	**TA100 Without S9**		**TA100 with S9**	
	**Fold Increase**	**Significant?**	**Fold Increase**	**Significant?**
Baseline	1.00	No	1.00	No
Pos CTR	2.01	Yes	4.76	Yes
125 μM	0.56	No	0.57	No
100 μM	0.55	No	0.75	No
75 μM	0.71	No	0.54	No
50 μM	0.65	No	0.57	No
25 μM	0.91	No	0.40	No
	**TA102 Without S9**		**TA102 with S9**	
	**Fold Increase**	**Significant?**	**Fold Increase**	**Significant?**
Baseline	1.00	No	1.00	No
Pos CTR	2.01	Yes	2.00	Yes
125 μM	1.11	No	1.09	No
100 μM	1.09	No	1.08	No
75 μM	0.75	No	0.91	No
50 μM	0.85	No	1.08	No
25 μM	1.18	No	1.14	No

**Table 5:** Results obtained to determine the irritant potential of cannabinoids.

**Evaluation of Primary and Accumulated Dermal Irritability and Skin Sensitization**	
Total number of participants	58
∑ skin reactions (Idil)	0.00
Average skin irritation index (Mdil)	0.00
**Evaluation of Phototoxicity and Cutaneous Photosensitization**	
Total number of participants	30
∑ skin reactions (Idil)	0.00
Average skin irritation index (Mdil)	0.00
**Final result**	Non-irritating—very good skin compatibility

**Table 6:** Results of the inhibition of enzymes with cosmetic relevance. Average inhibition percentage results are expressed as mean ± SD where *n* = 3.

		Average Inhibition Percentage (%)		
		MMP1	Elastase	Tyrosinase
Inhibition CTR		92.71	100.0	98.93
Solvent CTR		3.760	4.190	0.5800
CBG	100 μM	45.17	57.59	86.84
	50 μM	35.32	23.95	24.88
	10 μM	55.20	25.72	3.85
	5 μM	59.29	16.69	35.75
CBD	100 μM	49.81	30.83	24.25
	50 μM	67.47	13.87	38.36
	10 μM	14.87	20.36	8.670
	5 μM	41.64	37.56	30.34

**Table 7:** Skin permeation results of CBD and CBG assessed through mass balance (%) after 24 h.

		Mass Balance (%)			Absorbed Dose (μg/cm^2^)
		Dislodgeable Dose	Skin	Absorbed Dose	
CBD	1n	4.150 ± 0.78	25.50 ± 0.710	70.35 ± 1.49	514.6 ± 34.3
	2n	1.450 ± 0.21	32.00 ± 1.41	66.55 ± 1.63	512.5 ± 2.26
CBG	1n	1.800 ± 0.14	28.85 ± 9.69	69.35 ± 9.83	477.6 ± 37.2
	2n	3.350 ± 1.91	26.19 ± 5.39	70.46 ± 3.48	569.2 ± 26.7

## Data Availability

The original contributions presented in the study are included in the article/Supplementary Material, further inquiries can be directed to the corresponding author/s.

## Supplementary Materials

The following supporting information can be downloaded at: https://www.mdpi.com/article/10.3390/ijms252212224/s1. Refs. [58,59,60] are cited in the Supplementary Materials.
