# Supplementary material for: Exploring Cannabidiol (CBD) and Cannabigerol (CBG) Safety Profile and Skincare Potential
Source: Int J Mol Sci. 2024 Nov 14;25(22):12224. doi: 10.3390/ijms252212224 (PMC11595262; doi:10.3390/ijms252212224)
Supplement: Supplementary file 1 [file ijms-25-12224-s001.zip › ijms-3293685-supplementary.pdf]

International Journal of
Molecular Sciences

MDPI

A1 - Supplementary materials and methods

Supplementary Table S1. Primers sequences used for qPCR.

Gene	Forward primer 5′ -> 3′	Reverse primer 3′ –> 5′
18S rRNA (*18S1*)	CGTCTGCCCTATCAACTTTC	TTTTCGTCACTACCTCCCC
Actin Beta (*ACTB*)	CATCGTCCACCGCAAATGCTTC	CTCTACGAGACTCAAGCTTGC
Tubulin Alpha 1a (*TUBA1A*)	TTTATGCCTGGCTTTGCCCCTC	GCTGCTGTTCTTGTTCTGCACG
Keratin 10 (*KRT10*)	AGTCCCAACTGGCCTTGAAAC	TGCACACAGTAGCGACCTTC
Keratin 14 (*KRT14*)	GATGGCAGAGAAGAACCGCAAG	TGCTTTCATGCTGAGCTGGGAC
Collagen type 1, alpha 1 chain (*COL1A1*)	CGAAGACATCCCACCAATCACC	TCATCGCACAACACCTTGCC
Collagen type 1, alpha 2 chain (*COL1A2*)	AAGGAGAGAGCGGTAACAAGGG	AGACCACGAGAACCAGGACTAC
Fibronectin (*FN1*)	TGTTTACCAACCGCAGCCTCAC	TGCACCAAAGATGTCCGTCCTG
Hyaluronan synthase 1 (*HAS1*)	AAGCGCGAGGTCATGTACACAG	ACAAGCCCGCTCCACATTGAAG
Aquaporin 3 (*AQ3*)	ACAGCCCCTTCAGGATTTCCAC	CACACATGCACACACATGCAC

Supplementary Table S2. Conditions associated with the dermal phototoxicity and photosensitization tests.

**Test area irradiated**	**1 cm²**
**Distance from the lamp**	40 cm
**Lamp output**	4.57 mW/cm²/-0.013 DEM/min
**Total irradiation**	16-20 J/cm²
**UVA+UVB radiation time**	4 min per irradiation
**UVA+UVB total irradiation**	6-8 J/cm²
**UVA radiation time**	Approximately 20 minutes (1 irradiation)
**Total irradiation UVA**	5-7 J/cm²

The scale used to read reactions was recommended by the International Contact Dermatitis Research Group (IRCDG). The evaluation parameters were erythema, edema, papules, and vesicles. The skin irritation index was calculated based on the scores assigned to adverse reactions as shown in Supplementary Table S3.

Supplementary Table S3. Evaluation parameters for reading reactions.

Reactions	Results	Score
No reaction	Negative (-)	0
Doubtful reaction, mild erythema without definition borders	Doubtful (+/-)	0.5
Clear erythema – Presence of homogeneous erythema, edema, possible papules, and traces of vesicles.	Positive (+))	1
Presence of erythema and edema, papules, and medium-sized vesicles that possibly extravasate the area of application.	Positive (++)	2
Presence of erythema and edema, appearance of papules and vesicles of considerable size, and in some cases, blistering.	Positive (+++)	3

The evaluation results obtained at the different evaluation times were calculated to obtain the Individual Skin Irritation Index (IdiI). This value corresponds to the sum of all

individual scores obtained for each reaction observed at all reading times. The Mean Skin Irritation Index (MdiI) was calculated according to the following formula:

$$Mdil = \frac{\sum(Idil)}{Number\ of\ research\ participants}$$

The irritation index obtained allowed classifying the product according to the following scale:

Supplementary Table S4 – Classification of skin irritation potential.

Mdil	Results
0.0	Non-irritating – Very good skin compatibility
0.2	Non-irritating – Good skin compatibility
0.2 – 0.5	Slightly irritating – Regular skin compatibility
0.5 – 1	Moderately irritating – Poor skin compatibility
> 1	Irritant – Very poor skin compatibility

A2 - Supplementary results

Cannabinoids toxicity against TK-6 and THP-1 was also assessed (Supplementary Figure S1), with cell viability being over 50% for concentrations as high as 50 µM for both cell lines. Therefore, all tested cannabinoids were considered safe at 10 µM, establishing this as the concentration to be used in future in vitro assays.

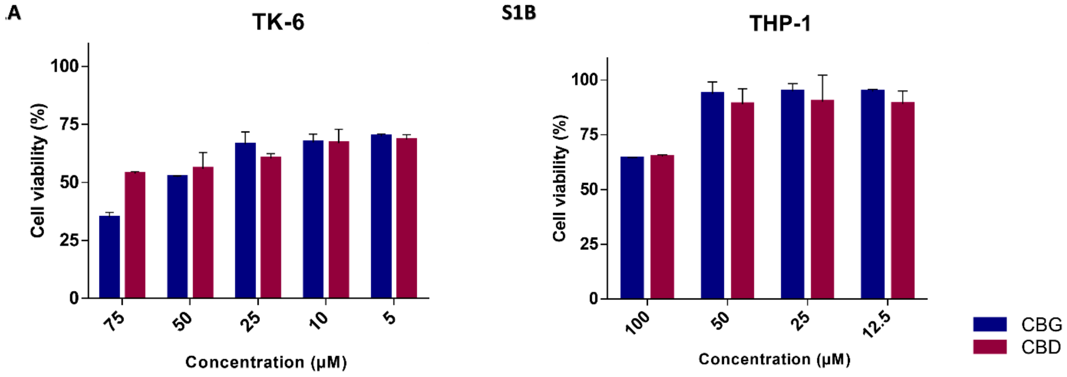

Supplementary Figure S1 - Results for the cell viability assay of TK-6 (**A**) and THP-1 (**B**) after exposure to cannabinoids. Data represented as mean ± SD for n = 3 independent experiments.

The micronucleus assay was performed using flow cytometry. Representative images are shown in Supplementary Figure S2, with a clear representation of micronucleus in the presence of the positive control, mitomycin (Supplementary Figure S2B).

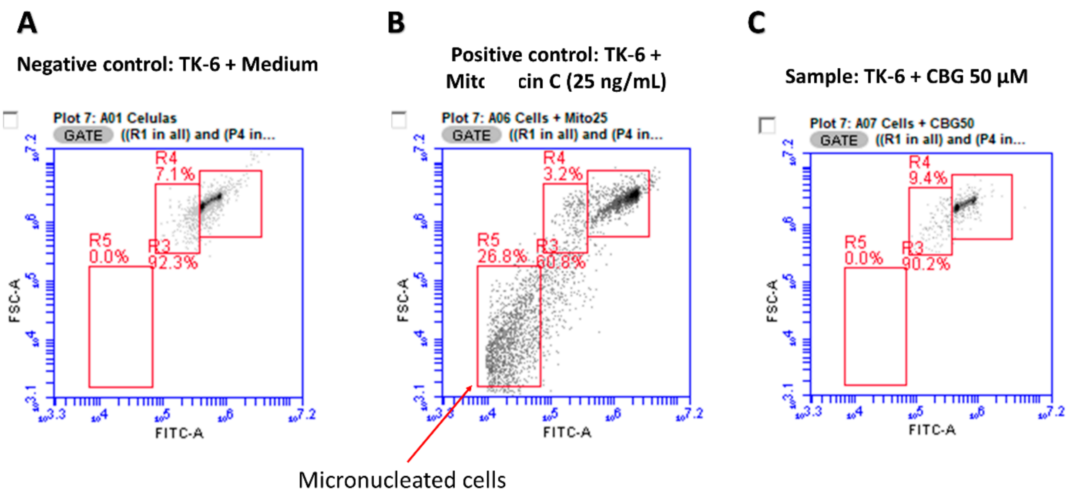

Supplementary Figure S2 - Representative results of the micronuclei assay. (**A**) Cells with medium only (negative control) led to 0% of micronucleated cells. (**B**) Mitomycin C (positive control) led to an average of 30% of micronucleated cells. Micronucleated cells are indicated with a red arrow. (**C**) CBG at the highest concentration did not result in the development of micronucleus.

Besides collagen, fibronectin levels were also assessed through different methodologies. Results showed a 15-fold increase in gene expression (Supplementary Figure S3) at the highest concentration tested (10 µM). However, these results did not translate into protein expression, as no changes in the production of fibronectin were verified for none of the compounds tested (Supplementary Figure S3B and C).

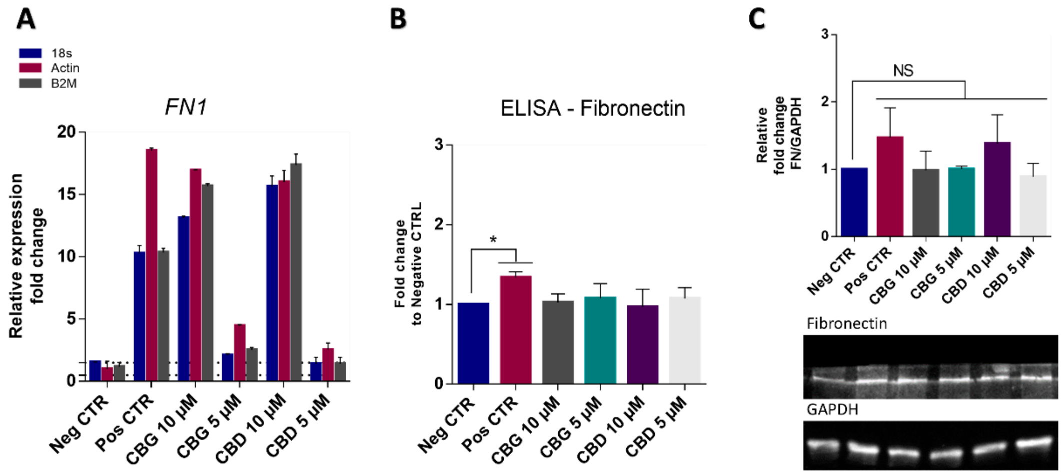

Supplementary Figure S3 - Cannabinoids did not impact the expression of fibronectin in human dermal fibroblasts. Results of qPCR with *FN1* (**A**), using three housekeeping genes show a significant ($p < 0.01$) increase for CBG and CBD at 10 µM. Fibronectin levels were also assessed by ELISA (**B**) and Western blot (**C**). No statistical differences were found between the negative control and the cannabinoid samples for these assays. Pal-GHK was used as a positive control (Pos CTR). Data were obtained from three independent experiments with two replicates for each condition. * $p < 0.05$, determined by one-way ANOVA coupled with Tukey's post hoc test.

In addition to collagen and fibronectin, other skin care markers were assessed in vitro. This included aquaporin 3, to study hydration; elastin, to assess the prevention of wrinkles; and hyaluronic acid (HA), a glycosaminoglycan (GAG) present in the ECM that

also contributes to the normal integrity of the skin [58–60]. As can be seen in Supplementary Figure S4, only the genetic expression of elastin was increased in the presence of CBD and CBG, with no changes verified for the remaining markers assessed.

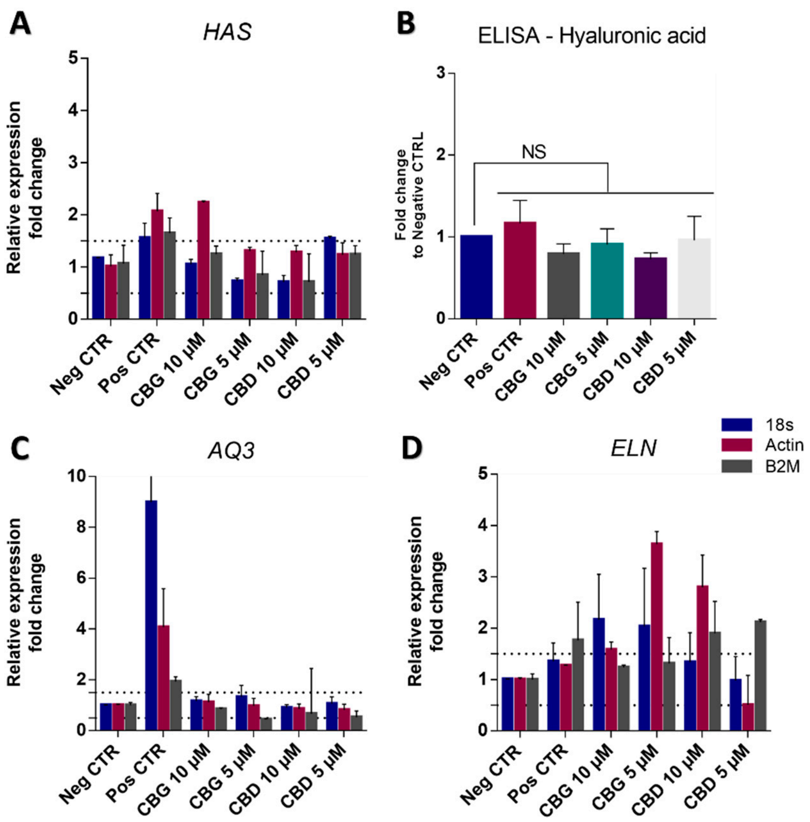

Supplementary Figure S4 - Results for qPCR with *HAS* (**A**), *AQ3* (**C**), and *ELN* (**D**) with three housekeeping genes. Hyaluronic acid levels were also assessed by ELISA (**B**). Pal-GHK was used as a positive control (Pos CTR). Data were obtained from three independent experiments with two replicates for each condition. No statistical differences (NS) were found between the negative control and the cannabinoid samples, determined by one-way ANOVA coupled with Tukey's post hoc test.
